# Tracking the carbon flows in municipal waste management in China

**DOI:** 10.1038/s41598-024-51698-0

**Published:** 2024-01-17

**Authors:** Jing Zhang, Huanzheng Du, Tao Wang, Peiyuan Xiao, Sha Lu, Gang Zhao, Jianfu Zhao, Guangming Li

**Affiliations:** 1 Circular Economy Research Institute, School of Marxism, Tongji University, 1239 Siping Rd., Shanghai, 200092 China; 2 Institute of Carbon Neutrality, Tongji University, 1239 Siping Rd., Shanghai, 200092 China; 3 College of Environmental Science and Engineering, Tongji University, 1239 Siping Rd., Shanghai, 200092 China; 4 UNEP-Tongji Institute of Environment for Sustainable Development, Tongji University, 1239 Siping Rd., Shanghai, 200092 China; 5 Shanghai Urban Construction Design & Research Institute Groups Co., Ltd., 3447 Dongfang Rd., Shanghai, 200120 China

**Keywords:** Climate-change impacts, Sustainability

## Abstract

Municipal solid waste (MSW), a carbon-intensive waste stream, may create both instant and indirect impacts onto environmental and climate management. Despite multiple studies made for greenhouse gases (GHGs) emissions of municipal waste, this research aims to achieve a comprehensive assessment for the carbon cycle by exploring evolution of waste composition and temporal-spatial disparities in waste management. Carbon flows embodied in MSW have been estimated across 31 provinces in Mainland China in the period 2000–2018. This improved estimation could be 15–40% smaller than the conventional estimation employing a constant waste composition. Aggregately some 578 ± 117 megatonnes carbon (MtC) were contained in MSW, including 239 ± 60 Mt of fossil carbon and 339 ± 58 Mt of degradable organic carbon. After treatment, 299 ± 66 MtC were possibly deposited in landfills and dumps. 279 ± 51 MtC were released to the atmosphere, creating net GHGs emissions equivalent to1870 ± 334 megatonnes of CO_2_ (MtCO_2_e). MSW generation in China nearly doubled during the period, net GHGs emissions increased by 1.8×, whereas fossil carbon grew by a factor of 3.5, mainly propelled by an increasing content of waste plastic in MSW. More rapid growth was witnessed in provinces in southern China than in northern. Distinct spatial–temporal evolution of waste and carbon metabolism was driven by increment, composition, and management effects. In the long run, the increment and composition effects may drop off. Enhanced practices of waste management integrating the circular economy are needed to fully recycle carbon flows, minimize emissions, and manage carbon deposits in aging landfills and dumps.

## Introduction

Nearly two gigatonnes of municipal solid waste (MSW) are annually generated in the world, in which two-thirds are from the developing countries^1^. The world’s largest MSW producer as from 2004 is China. Its waste generation climbed from 118 megatonnes (Mt) in 2000 to 242 Mt in 2019^2,3^. Safe management of municipal waste is one of the most pressing environmental challenges faced by many millions of low and middle incomes^1^.

MSW has profound implications for climate change. It contains a big proportion of food waste and an increasing content of waste paper and plastic^1^. These carbon-rich compositions can be converted into carbon dioxide (CO_2_) and methane (CH_4_) in the process of waste management^4^. CO_2_ from fossil sources (e.g., plastic and rubber) and CH_4_ can cause extra global warming, and thus are defined by the Intergovernmental Panel on Climate Change (IPCC) as net greenhouse gases (GHGs) emissions. CO_2_ from biological sources, however, is normally treated as climate neutral. Additionally, there is a quantity of carbon resistant to degradation and deposited in landfills and dumps. Aging infrastructure and poor management of carbon deposit may provoke chronical or abrupt risks to public health and climate. In order to limit global warming within 1.5 °C, net zero emissions and carbon neutrality are to be accomplished by mid-twenty-first century^5^. Many countries, including those in the European Union, Japan, South Korea, and U.K. have set their targets of carbon or climate neutrality^6^. China also pledges to achieve peak CO_2_ emissions before 2030 and to be carbon neutral by 2060^7^. Emissions from waste disposal should be substantially abated to fulfill the climate targets.

A body of research has been carried out to assess GHGs emissions from municipal waste and to explore mitigation strategies. Whereas sampling and analytical techniques can provide direct and more precise measurement of emissions^8,9^, regional- and country-level assessment mainly relies on modelling, especially the mass balance method from IPCC’s guidelines^10^. For GHGs from MSW in China, Wang et al.^11^ calculated net emissions in multiple provinces from 2003 to 2012. The rate of tertiary industry in GDP and the urban per capita disposable income were found having an indirect positive effect on the emissions. Zhao et al.^12^ evaluated the emissions in seven regions in China and noted that methane from landfills contributed most emissions, thus methane mitigation technologies such as landfill gas to energy projects and functional solid cover were recommended. Du et al.^13^ estimated methane emissions in MSW landfills by using the first-order decay (FOD) model. Methane emissions were strongly and positively correlated with population and socioeconomic demographics. Liu et al.^4^ compared five scenarios of waste and carbon management involving landfill with gas recovery, incineration, and biological treatment. Landfill gas collection systems were inadequately equipped or operated in many landfills, making landfill gas release a noted source of GHGs emissions^14,15^.

GHGs emissions caused by MSW were also assessed for a series of cities and provinces in China, e.g., Beijing, Shanghai, Guangzhou, Dongguan, Hangzhou, and Lhasa^16–21^. Most research employed a similar mass balance approach. Nevertheless, waste composition and moisture in MSW were demonstrated changing from one place to another, leading to different emission patterns. The spatial difference in waster property and management were also illustrated by international and inter-regional comparative studies. Mühle et al.^22^ detected that carbon emissions in the U.K. could be five times greater than those in Germany to treat the same quantity of MSW, because the landfilling rate is higher and energy recovery from waste is lower in the former. Ding et al.^23^ compared MSW management between cities in China and the intentional counterparts including Berlin, Singapore, and Tokyo. In contrast to the world’s highly developed cities, MSW in Chinese contained higher organic content and generated more GHGs. Transfer of advanced and emerging technologies for resource utilization from leading to less developed countries can create marked benefits for climate mitigation and the urban environment^24^.

Overall, the MSW property and management practices were found performing a wide diversity, especially in a country as big, heterogenous, and fast changing as China^25,26^. Multiple studies had been conducted to assess the global warming potential of municipal waste in the country as a whole or in specific cities^27^. These studies mainly centered on GHGs emissions and their waste causes. A truly comprehensive assessment of carbon metabolism of MSW is still needed. This present research aims to examine the municipal waste and carbon flows in all provincial administrative divisions in China over a longer period. MSW composition has been evaluated for as many places and time as possible, and socio-economic drivers to waste property and management explored. We also attempt to seek more inclusive strategies to tackle waste management and climate change.

## Methods and data

This research aims to assess municipal waste flows and carbon content embodied in MSW across 31 provinces in Mainland China in the period 2000–2018. A material flow analysis was carried out from waste generation to treatment. Indirect carbon emissions caused by energy use of waste collection and transportation were not included in the system boundary. Illegal dumping and informal recycling were not counted neither, because dumping was restricted in the new century^28^. The informal sector could be active in collecting, processing, and trading of recyclable materials, but the quantity and composition of recyclables informally picked out of the municipal waste streams can hardly be tracked province by province due to data limitations^29–31^.

### Waste composition

Nine components in MSW can be identified, i.e., food waste, plastic, textile, paper, metal, wood, stone, glass, and others. Degradable organic carbon (DOC) and fossil carbon content mainly exist in food waste, plastic, textile, paper, and wood^11^. Their composition in the gross weight of MSW are presented in Table 1. The moisture content in MSW ranges from 36 to 66% for different components (See the supplementary file for details). For DOC and fossil carbon content in waste dry weight, the default values of the IPCC^32^ guidelines are applied (See the supplementary file for details).

**Table 1:** Composition of MSW in China, mean ± standard deviation of 184 waste samples from 50 cities.

Year	Food waste %	Plastic %	Wood %	Paper %	Textile %
1990s	65.4 ± 16.8	9.8 ± 3. 6	1.7 ± 0.8	6.5 ± 2.9	2.4 ± 1.1
2000s	50.1 ± 10.4	13.0 ± 4.5	2.5 ± 2.3	8.7 ± 4.4	3.7 ± 2.9
2010s	50.3 ± 12.6	13. 9 ± 4.5	4.0 ± 3.9	12.8 ± 4.0	3.2 ± 2.9

### Carbon flows

Carbon coming from paper, textile, food waste, wood, and plastic are computed by Eq. (1). $TC$ represents the total carbon content in MSW. ${\text{W}}$ is the gross weight of MSW. ${c}_{t}$ is the total carbon content in gross MSW. ${\alpha }_{i}$ represents the percentage of component *i* in gross MSW. ${v}_{i}$ represents the moisture content of composition *i*. ${c}_{t,i}$ represents the carbon content in the dry weight of component *i*.

1 $${\text{TC}}={\text{W}}\times \sum_{i}{\alpha }_{i}\times (1-{v}_{i})\times {c}_{t,i}$$

The DOC flows are calculated by Eq. (2). $DOC$ represents the degradable organic carbon flows in MSW. ${c}_{o}$ is the aggregate DOC percentage in gross MSW. ${c}_{o,i}$ represents the DOC percentage in the dry weight of component *i*. ${\text{W}}$_,_ ${\alpha }_{i}$, and ${v}_{i}$ are defined in Eq. (1).

2 $${\text{DOC}}={\text{W}}\times \sum_{i}{\alpha }_{i}\times (1-{v}_{i})\times {c}_{o,i}$$

The fossil carbon flows are computed by Eq. (3). $FoC$ represents the fossil carbon content in MSW. ${c}_{f}$ is the fossil carbon content in gross MSW. ${c}_{f,i}$ represents the fossil carbon content in the dry weight of component *i*. ${\text{W}}$_,_ ${\alpha }_{i}$, and ${v}_{i}$ are defined in Eq. (1).

3 $${\text{FoC}}={\text{W}}\times \sum_{i}{\alpha }_{i}\times (1-{v}_{i})\times {c}_{f,i}$$

### GHGs emissions

GHGs emissions from incineration, composting, sanitary landfills, and simple landfills are estimated as below. More details about the computation are illustrated in the supplementary file.

#### Sanitary landfills

GHGs emissions from landfills can be computed by two approaches, one relying on mass balance and another employing the FOD model^33^. More accurate FOD estimation requires time series landfilling information as long as 50 years, which can hardly be obtained for hundreds of landfills in China. The mass balance method is therefore adopted in this research, and historic waste compositions are considered to mitigate the possible overestimation of emissions.

${E}_{L,{\text{CH4}}}$ is CH_4_ emissions, and ${E}_{L,{\text{CO2}}}$ is CO_2_ emissions from sanitary landfills. *W*_*L*_ is the amount of MSW landfilled. ${dc}_{L}$ is decomposed proportion of DOC during landfills. ${mcf}_{L}$ is methane correction factors of sanitary landfills. ${\mu }_{L}$ is the proportion of CH_4_ in landfills gas by volume. $ox$ is methane oxidation factor in landfills. 12, 16, 44 are molecular weight of carbon, CH_4_, and CO_2_, respectively. CH_4_ has a global warming potential some 25 times greater than that of CO_2_. ${{\text{c}}}_{{\text{o}}}$ is the aggregate DOC percentage in gross MSW. ${E}_{L,{\text{CH4}},REC}$ is the recovery amount of CH_4_ in landfills. ${E}_{L,{\text{CH4}},FLA}$ is the flaring amount of CH_4_ in landfills. ${E}_{L,{\text{CH4}},REL}$ is the released amount of CH_4_ in landfills. ${r}_{{\text{CH4}}}$ is landfills gas recovery ratio. ${f}_{{\text{CH4}}}$ is landfills gas flaring ratio. The computation is as follows:

4 $${E}_{L,{\text{CH4}}}={W}_{L}\times {c}_{o}\times {dc}_{L}\times {mcf}_{L}\times {\mu }_{L}\times (1-ox)\times \frac{16}{12}$$

5 $${E}_{L,{\text{CH4}},REC}{=E}_{L,{\text{CH4}}}\times {r}_{{\text{CH4}}}$$

6 $${E}_{L,{\text{CH4}},FLA}{=E}_{L,{\text{CH4}}}\times {f}_{{\text{CH4}}}$$

7 $${E}_{L,{\text{CH4}},REL}{=E}_{L,{\text{CH4}}}-{E}_{L,{\text{CH4}},REC} -{E}_{L,{\text{CH4}},FLA}$$

8 $${E}_{L,{\text{CO2}}}={W}_{L}\times {c}_{o}\times {dc}_{L}\times {[1-mcf}_{L}\times {\mu }_{L}\times (1-ox)]\times \frac{44}{12}+{E}_{L,{\text{CH4}},FLA}\times \frac{44}{16}$$

#### Incineration

CO_2_ emissions from MSW incineration are computed in Eq. (9). ${E}_{I}$ represents emissions from incineration. ${W}_{I}$ is the MSW amount for incineration. $\delta$ is oxidation factor in incineration. ${c}_{t}$ is the total carbon content in gross MSW.

9 $${E}_{I}={W}_{I}\times {c}_{t}\times \delta \times \frac{44}{12}$$

#### Composting

The biodegrade carbon mainly turns to CO_2_ after composting. The emissions are estimated in Eq. (10) to Eq. (11). ${E}_{C,{\text{CO}}2}$ represents CO_2_ emissions from composting. ${E}_{C,{\text{CH}}4}$ represents CH_4_ emissions from composting. ${W}_{C}$ is composting amount of MSW. ${dc}_{C}$ is the decomposed proportion of DOC during composting. ${dc}_{{\text{CO}}2}$ is the decomposed proportion of CO_2_ during composting. ${dc}_{{\text{CH}}4}$ is the decomposed proportion of CH_4_ during composting. ${c}_{o}$ is the aggregate DOC percentage in gross MSW.

10 $${E}_{C,{\text{CO2}}}={W}_{C}\times {c}_{o}\times {dc}_{{\text{CO2}}}\times \frac{44}{12}$$

11 $${E}_{C,{\text{CH4}}}={W}_{C}\times {c}_{o}\times {dc}_{{\text{CH4}}}\times \frac{16}{12}$$

#### Simple landfills and dumping

GHGs emissions from simple landfills are calculated in Eq. (12) to Eq. (13). Emissions from dumps are assumed following the same computation. ${E}_{D,{\text{CH4}}}$ represents CH_4_ emissions and ${E}_{D,{\text{CO}}2}$ represents CO_2_ emissions from simple landfills and dumping. ${W}_{D}$ is the amount MSW entering simple landfills and dumps. ${dc}_{D}$ is decomposed proportion of DOC in simple landfills and dumps. ${mcf}_{D}$ is methane correction factors of simple landfilling and dumping. ${\mu }_{D}$ is the proportion of CH_4_ in landfill and dumping gas. ${c}_{o}$ is the aggregate DOC percentage in gross MSW.

12 $${E}_{D,{\text{CH}}4}={W}_{D}\times {c}_{o}\times {dc}_{D}\times {mcf}_{D}\times {\mu }_{D}\times \frac{16}{12}$$

13 $${E}_{D,{\text{CO}}2}={W}_{D}\times {c}_{o}\times {dc}_{D}\times (1-{mcf}_{D}\times {\mu }_{D})\times \frac{44}{12}$$

#### Net GHGs emissions

Net GHGs emissions include CO_2_ from burning of fossil carbon and CH_4_ from landfills and dumps, as computed in Eq. (14). ${E}_{n}$ represents net GHGs emissions. ${E}_{L,{\text{CH4}},REL}$ is the released amount of CH_4_ in landfills. ${E}_{D,{\text{CH4}}}$ represents CH_4_ emissions from dumping. ${E}_{C,{\text{CH4}}}$ represents CH_4_ emissions from composting. ${W}_{I}$ is the MSW amount for incineration. ${c}_{f}$ is the fossil carbon content in gross MSW. $\delta$ is oxidation factor in incineration.

14 $${E}_{n}=25\times ({E}_{L,{\text{CH4}},{\text{REL}}}+{E}_{D,{\text{CH4}}}+{E}_{C,{\text{CH4}}})+{W}_{I}\times {c}_{f}\times \delta \times \frac{44}{12}$$

CO_2_ originating from biological content can be treated as carbon neutral. The quantity of neutral emissions from MSW can be estimated by Eq. (15). ${E}_{u}$ represents neutral GHGs emissions. ${E}_{L,{\text{CO2}}}$ is CO_2_ emissions from sanitary landfills. ${W}_{I}$ is the MSW amount for incineration. ${c}_{o}$ is the aggregate DOC percentage in gross MSW. $\delta$ is oxidation factor in incineration. ${E}_{C,{\text{CO2}}}$ represents CO_2_ emissions from composting. ${E}_{D,{\text{CO2}}}$ represents CO_2_ emissions from simple landfills and dumping. ${E}_{L,{\text{CH4}},REC}$ is the recovery amount of CH_4_ in landfills.

15 $${E}_{u}={E}_{L, {\text{CO2}}}+{W}_{I}\times {c}_{o}\times \delta \times \frac{44}{12}+{E}_{C,{\text{CO2}}}+{E}_{D,{\text{CO2}}}+{E}_{L,{\text{CH4}},REC}\times \frac{44}{16}$$

The total emissions are simply the sum of net plus neutral emissions.

16 $${E}_{tot}={E}_{n}+{E}_{u}$$

### Carbon deposit

The remaining carbon content may be accumulated in landfills and dumps, difficult to be decomposed. The amount of carbon deposit can be estimated by the mass balance of total carbon minus air released carbon, as presented in Eq. (17). $DeC$ represents deposited carbon. $TC$ represents the total carbon content in MSW. ${E}_{L,{\text{CH}}4,REL}$ is the released amount of CH_4_ in landfills. ${E}_{L,{\text{CH}}4,REC}$ is the recovery amount of CH_4_ in landfills. ${E}_{D,{\text{CH}}4}$ represents CH_4_ emissions from dumping. ${E}_{C,{\text{CH}}4}$ represents CH_4_ emissions from composting. ${E}_{L,{\text{CO}}2}$ is CO_2_ emissions from sanitary landfills. ${E}_{I}$ represents emissions from incineration. ${E}_{C,{\text{CO}}2}$ represents CO_2_ emissions from composting. ${E}_{D,{\text{CO}}2}$ represents CO_2_ emissions from simple landfills and dumping.

17 $$ \begin{aligned} DeC = & TC - \left( {E_{{L,{\text{CH}}4,REL}} E_{{L,{\text{CH}}4,REC}} + E_{{D,{\text{CH}}4}} + E_{{C,{\text{CH}}4}} } \right) \times \frac{12}{{16}} \\ & - \left( {E_{{L,{\text{CO}}2}} + E_{I} + E_{{C,{\text{CO}}2}} + E_{{D,{\text{CO}}2}} } \right) \times \frac{12}{{44}} \\ \end{aligned} $$

## Results

### Modelling of carbon content

**Figure 1:**
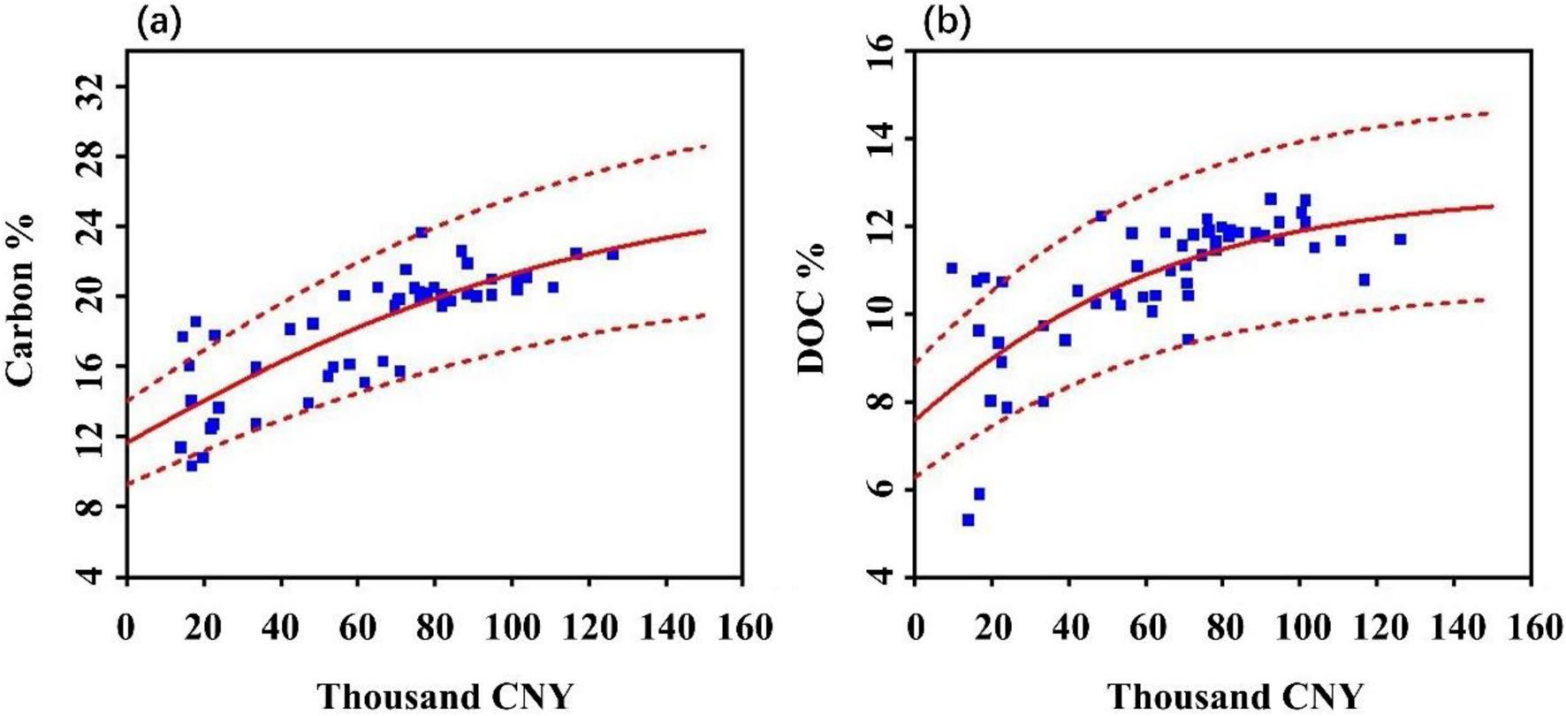
Regression of carbon content in MSW versus GDP per capita. (**a**) Total carbon, and (**b**) DOC. The dashed lines indicate a confidence interval of 90%.

A dataset of MSW composition was developed, containing 184 samples from 50 Chinese cities in 1990–2018 (See the supplementary file for details). With this dataset, we attempted to identify the relationship between carbon content in MSW and its influential factors from personal income, living expenses, and retail sales of consumer goods (as shown in the supplementary file). Multiple regressions had been testified, and a sigmoidal growth curve to GDP per capita was proved as more notable and ease of use (Fig. 1). The content of total carbon and DOC increased and finally reached a plateau with the income growth. The corresponding SGompertz model and parameters are displayed in the following equation.

18 $$c=a\times {e}^{{-e}^{-k(x-{x}_{c})}}$$

*c* in Eq. (16) represents carbon content in MSW. *x* is GDP per capita. *a* is an asymptote when *x* tends to infinity. ${x}_{c}$ defines the center and *k* sets the growth rate of the curve.

The regression results are presented in Fig. 1. The most fitted estimate is that *a* = 26.796, *x*_*c*_ = − 1.4218 and *k* = 0.1280 for total carbon, and *a* = 12.797, *x*_*c*_ = − 3.2905 and *k* = 0.1961 for DOC. The uncertainty range of the parameters is also analyzed. The biggest impact may be caused by parameter *a*, which changes from 21.336 to 32.256 for TC and 10.607 to 14.987 for DOC under 90% confidence interval. More details about the regression are included in the supplementary file. This regression model was employed to approximate the spatial–temporal variation of carbon flows in provinces of China.

### Carbon flows in MSW

**Figure 2:**
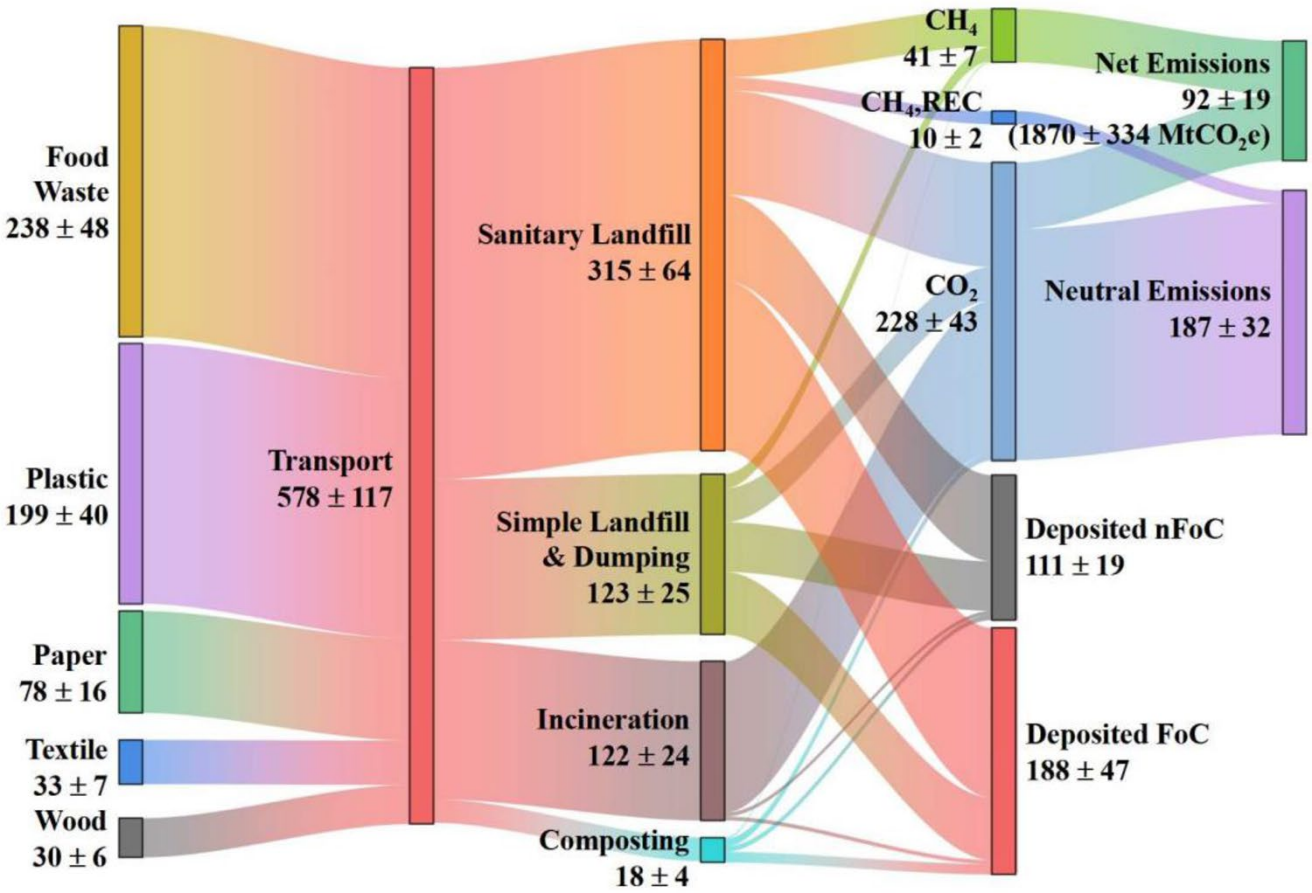
Cumulative carbon flows in MSW in China during 2000–2018, values are in MtC. FoC, fossil carbon; nFoC, non-fossil carbon.

The carbon content and flows throughout the municipal waste cycle were quantified for the 31 provinces in China. An aggregate of the country-level carbon metabolism during 2000–2018 is displayed in Fig. 2. The country-level carbon metabolism in 2000, 2010 and 2018 are displayed in Fig. S8–10 respectively in supplementary file. The values representing cumulative carbon flows are in megatonnes carbon (MtC). The figure portrays the carbon embodied in MSW only, indirect carbon emissions associated with MSW collection and transportation were not included.

In the past two decades, some 578 ± 117 MtC could be derived from MSW. About 41% of the carbon were contained in food waste, 21% in waste plastic, and 13% in waste paper. A majority of the carbon, 315 ± 64 MtC, was processed in sanitary landfills. The follows were 123 ± 25 MtC in simple landfills and dumps, 122 ± 24 MtC in incineration, and 18 ± 4 MtC by composting. After treatment, the mass balance computation estimated that approximate 279 ± 51 MtC were released to the atmosphere in the form of CO_2_ and CH_4_. The CO_2_ emissions from DOC and converted from landfill gas combustion were regarded as climate neutral, containing 279 ± 51 MtC. The remaining carbon flows were most likely deposited in landfills and dumps, including 188 ± 47 MtC of fossil carbon and 111 ± 19 MtC in non-fossil form.

### GHGs emissions

The carbon emissions from landfilling, incineration, composting, and dumping can be assessed with the equations above. From 2000 to 2018, net GHGs emissions including CH_4_ and CO_2_ from fossil content aggregately contained about 92 ± 19 MtC and generated global warming impact equivalent to 1870 ± 334 megatonnes of CO_2_ (MtCO_2_e). Annually, the net emissions in China increased from 67 ± 12 MtCO_2_e in 2000 to 130 ± 25 MtCO_2_e in 2018, rising by a factor of 1.9. With the widespread development of incinerators and increasing fossil carbon burned, emissions from incineration increased from < 1 to 38.9% of the total net GHGs emissions. Net emissions from sanitary landfills dropped from 68.9 to 59.2% in 2000–2018.

**Figure 3:**
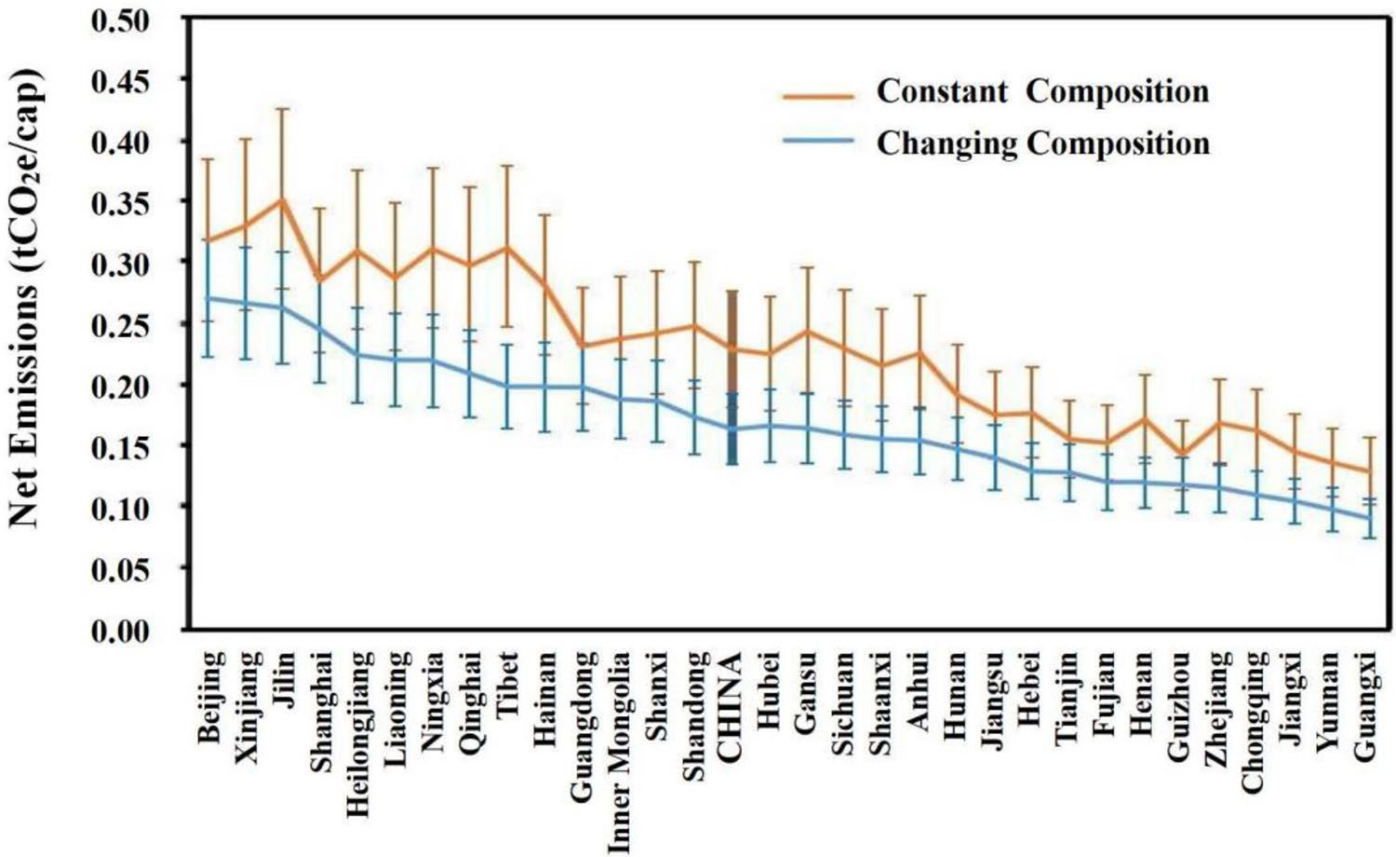
Estimation of net emissions per capita based upon constant and changing waste composition. Annually average per capita emissions in 2000–2018 are displayed for 31 provinces. The upper and lower whiskers define a confidence interval of 90%.

Our research found that the conventional approach employing constant composition^10^ might overestimate MSW’s carbon flows and GHGs emissions. By examining the temporal and spatial changes of waste composition and the influence of economic development, better estimation of carbon metabolism can be obtained. Averagely, per capita net emissions at provincial level could be 14% to 36% lower in our computation (Fig. 3). Gaps bigger than > 30% were mainly observed in provinces in western China, including Gansu, Chongqing, Sichuan, Guizhou, and Tibet, where the landfilling rate was greater than the national average. The difference between the two methods was shrinking over time.

## Spatial and temporal discrepancy of carbon metabolism in MSW

**Figure 4:**
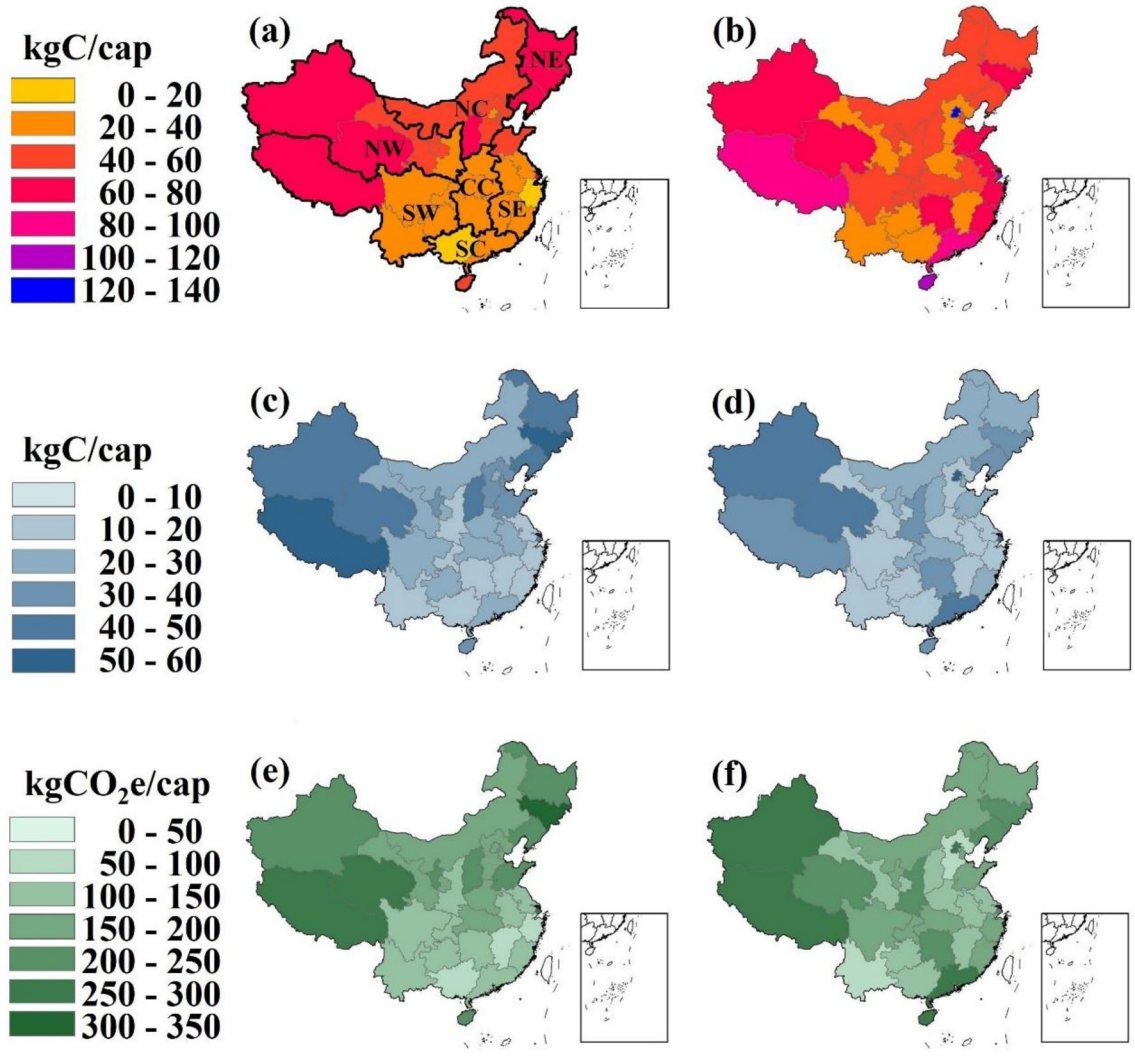
Per capita carbon flows in MSW in China, including total carbon per capita in (**a**) 2000, (**b**) 2018; deposited carbon per capita in (**c**) 2000, (**d**) 2018; and net GHGs emissions per capita in (**e**) 2000, (**f**) 2018. The maps are created by the authors with ArcGIS 10.8 (URL: https://www.esri.com/en-us/arcgis/products/arcgis-online/overview).

For a country as large and unevenly developed as China, it is no wonder to witness spatial disparities in municipal waste and carbon management. Figure 3 compares per capita flows of the total carbon content in MSW, carbon deposit increased in landfills and dumps, and net GHGs emissions in 2000 and 2018. The median carbon content in MSW across 31 provinces increased from 39.6 to 56.3 kgC/cap, but its standard deviation increased in a slower pace from 18.6 to 25.6 kgC/cap. For per capita net emissions, the median and standard deviation slightly decreased from 169 ± 66 to 162 ± 59 kgCO_2_e/cap. For per capita deposited carbon, the value changed from 26.9 ± 12.6 to 23.1 ± 12.7 kgC/cap. Overall, the regional discrepancy diminished in the past two decades. The most developed and densely settled areas, i.e., municipalities of Beijing and Shanghai, had the biggest per capita carbon and emissions flows. Nonetheless greater per capita flows were also seen in provinces in south- and west-most China including Qinghai, Xinjiang, Tibet, and Hainan, where both population density and economic development level are low. This suggest that the carbon flows might be affected by different driving forces (Fig. 4).

**Figure 5:**
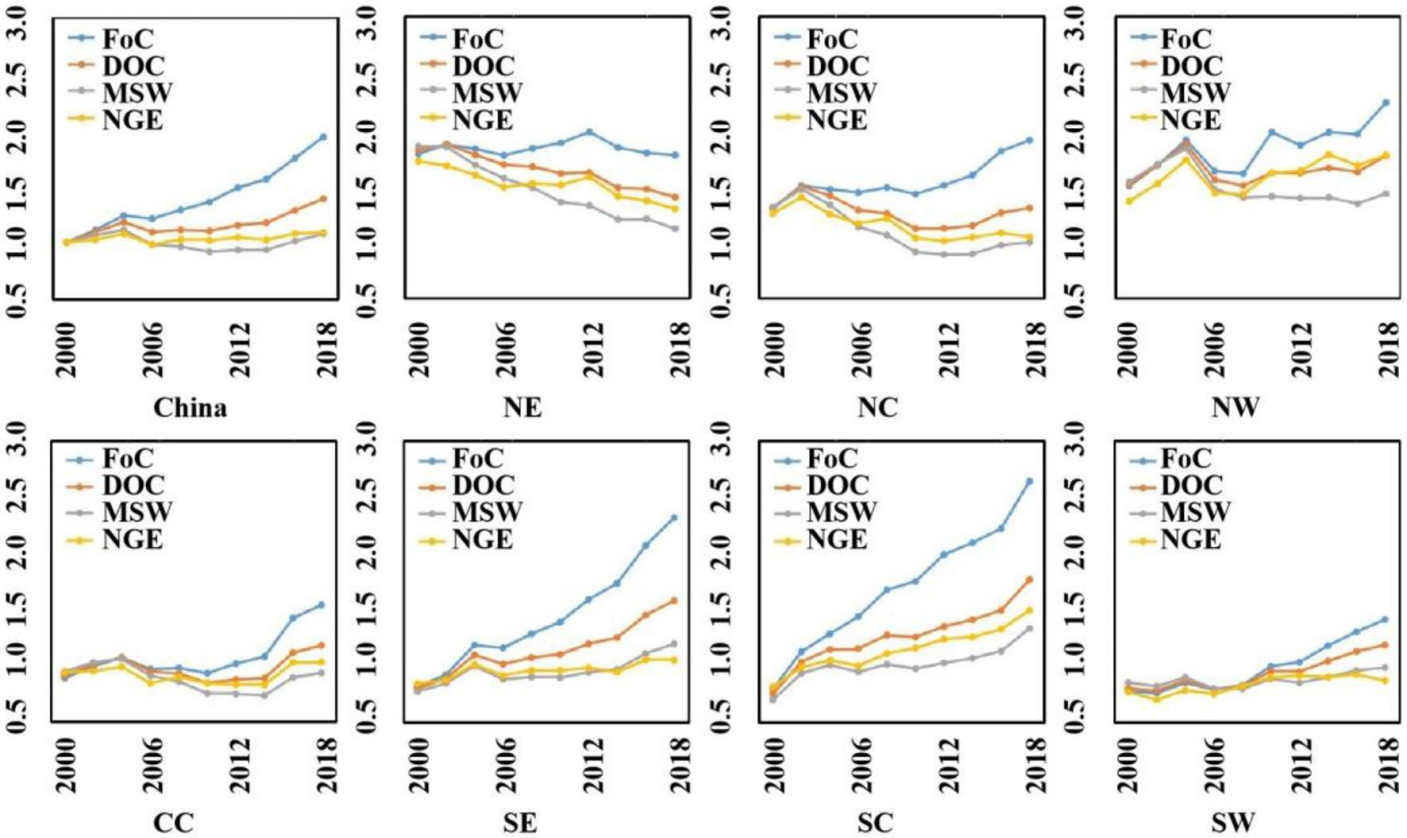
Change of per capita flows of MSW, fossil carbon (FoC), DOC, and net GHGs emissions (NGE), relative to per capita values of China in 2000. CC, Central China; NC, North China; NE, Northeast China; NW, Northwest China; SC, South China; SE, Southeast China; SW, Southwest China.

From an evolutionary perspective, we note that per capita MSW in the country increased by merely 7.5% during the period 2000–2018, per capita net GHGs emissions increased by 5.2%, per capita DOC in MSW grew by 38.6%, and a nearly double growth was seen from fossil carbon. The pattern that fossil carbon increased more rapidly than DOC, followed a sluggish change in net emissions and MSW generation is also applicable to most of the provinces and regions in China (Fig. 5). The fossil carbon flows were mainly driven by rapid increase of waste plastic in MSW. This is in line with the surge of e-commerce and food delivery and their rising demand for plastic packaging.

A spatial–temporal discrepancy can also be identified across the seven regions, i.e., Northeast China (NE), North China (NC), Northwest China (NW), Central China (CC), Southeast China (SE), South China (SC), and Southwest China (SW). The division of the regions are displayed in the map in Fig. 4. In the early 2000s, per capita flows in NE, NW, and NC were greater than those in CC, SE, SW, and SC. Nonetheless, SC and SE had led ahead the central and northern provinces in 2018, after a two-decade of strong growth in municipal waste and carbon in southern China versus swing or decrease in per capita flows in northern (Fig. 5). This southern-northern reverse can be interpreted by the following reasons. First, domestic coal stoves for home heating used to generate large amounts of coal ash, which could contribute a majority of municipal waste in winter in northern provinces (in NE, NC, NW, and partially CC). The ash, however, had been eliminated from MSW and combined into industrial waste after a massive substitution of central heating for old-fashioned stoves in the 2010s. This led to decline of MSW flows in the upper portion of China. In addition, faster increase of municipal waste and carbon flows in southern provinces could be attributed to its relative better performance in economic development and immigration. As early as 2000 there were six southern provinces among the top ten of economic output. The number had increased to eight in 2018.

## Discussion and conclusions

This research explored the carbon flows of MSW in China from a spatial–temporal evolutionary angle. Despite municipal waste becoming increasingly carbon intensive, GHGs emissions did not increase proportionally in the past two decades. The rate of net emissions per tonne of MSW climbed from 0.47–0.67 tCO_2_e in 2000 to 0.53–0.75 tCO_2_e in 2011, but thereafter shrank to 0.47–0.69 tCO_2_e in 2018. Our analysis suggested that net emissions could be 14% to 36% lower than the conventional estimation assuming a static waste composition (Fig. 3). More significant increase in waste and carbon flows were perceived in provinces and cities in southern areas than in northern. Nonetheless the spatial discrepancy had declined from a per capita basis (Fig. 5). The distinct spatial–temporal patterns of municipal waste and carbon metabolism could be interpreted by the following effects.

*Increment effect* indicates that urbanization and economic development could boost municipal waste generation. MSW kept increasing in China in 2000–2018, with an annualized rate of 3.7%. The increment was more significant in cities in South China (SC) and Southeast China (SE), where stronger performance in economic vitality and labor immigration was achieved. The trend likely maintains, as potential economic and urban growth is foreseeable in the near future.

*Composition effect* illuminates a rising carbon intensity (including both DOC and fossil carbon) in MSW. Higher standard of living can lead to use and discard of more carbon intensive materials. Nowadays in many cities, scrap paper and plastic have made some one-third of the municipal waste flows. Another noted composition change has occurred in coal ash from domestic heating. This carbon minimal waste, once constituting more than half of MSW in winter in northern China, has been eliminated after pervasion of central heating and substitution of natural gas boilers for coal-based system. The removal of coal ash from municipal waste management also promoted a decline of MSW in Northeast (NE), North (NC), and Northwest China (NW).

**Figure 6:**
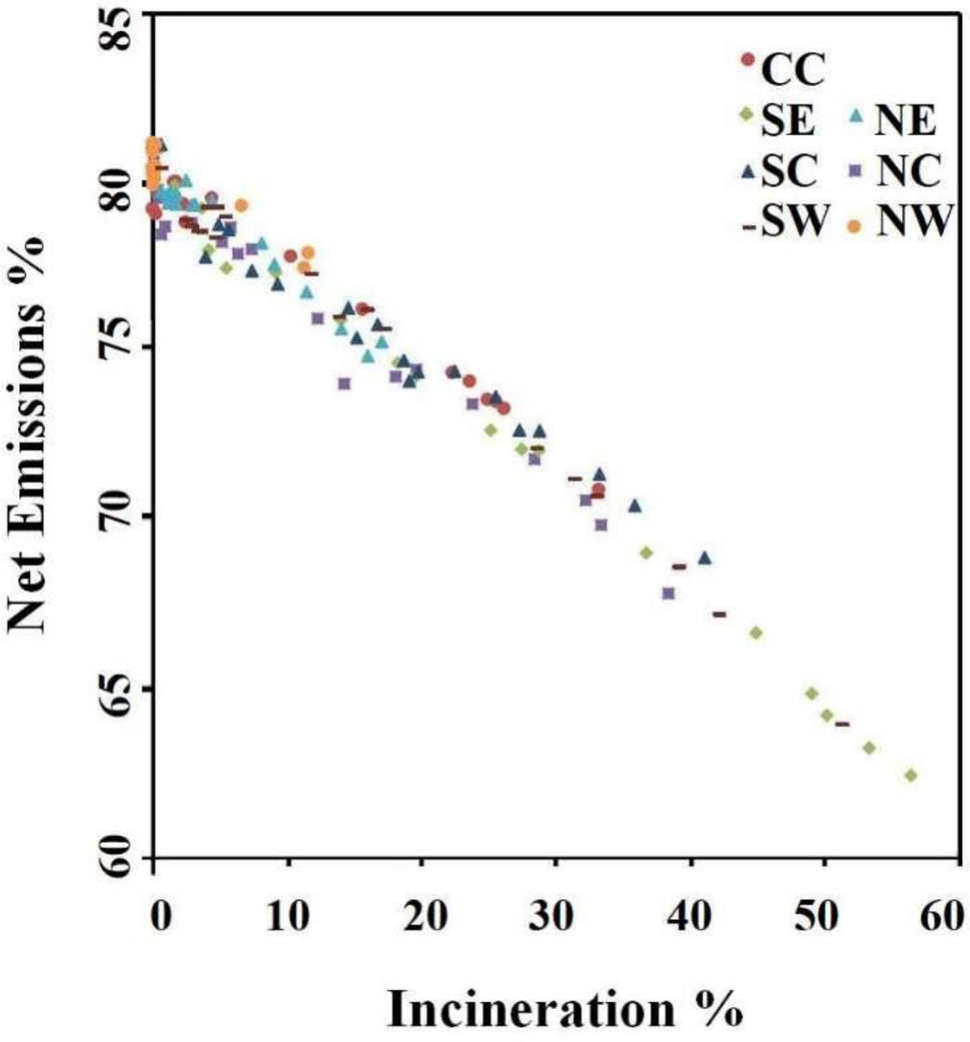
Relationship between incineration ratio and net GHG emission ratio. Incineration ratio is the percentage of MSW incineration to MSW generation. Net emission ratio is the quotient of *E*_*n*_ to *E*_*tot*_. CC, Central China; NC, North China; NE, Northeast China; NW, Northwest China; SC, South China; SE, Southeast China; SW, Southwest China.

*Management effect* curbs GHGs emissions with more environmentally friendly treatment of MSW. In the 2000s, > 80% of MSW might end up in sanitary or simple landfills^3^. Landfill gas collection and recycling systems were inadequately operated in many landfills^15^. Methane in landfill gas became a major source of net GHGs emissions, generating some 90% of the global warming potential. But today incineration has overtaken landfills as a mainstream treatment approach. The assessment showed that the net emission from incineration was 0.20–0.33 tCO_2_e per tonne of MSW treatment, lower than the landfill’s emission of 0.50–0.70 tCO_2_e/tMSW. The rate of net emission to total (net plus neutral) emission also declined with the proliferation of incineration (Fig. 6), suggesting that incineration was less climate damaging than landfills in China.

In summary, the present research offered insights into municipal waste flows and associated carbon emissions in China during 2000 to 2018. Our future work will keep tracking the changes in municipal waste and its management paradigm and global warming impact. It can be expected that the spatial disparity of waste flows will diminish in the long run. The increment and composition effects may become less influential, because both quantity and carbon intensity of MSW may reach a peak toward a highly developed and material circular society^14^.

On the other hand, the management effect will be likely dominant after some fundamental changes occurred in recent years. China launched a national initiative for waste sorting in 2017. Compulsory sorting of municipal waste into food waste, recyclables, hazardous waste, and residuals had been implemented or trialed in more than 80 cities in China as of 2020. The sorting system will be transplanted to all of the cities in 2025 to 2030^34^. Substantial investments had been pumped into facilities of waste sorting, food waste processing, and incineration. The number of incineration plants rocketed from 109 in 2011 to 648 in 2022. The incineration capacity alone reached 300 Mt per annum, already exceeding the municipal waste generation^35^.

Better waste management paradigm integrating waste sorting, material recycling, nutrient reclamation, energy recovery and incineration will substantially reform the present scheme of municipal waste and carbon metabolism. According to our analysis for the sorted waste in Shanghai, about 21% of the municipal streams were recyclables for material recycling. 32% were food waste from which energy and nutrients can be recovered by means of anaerobic digestion. The remaining 47% were mainly residual waste to be incinerated, and no more primary waste landfilled. Only the fossil carbon in residual waste can generate net emissions after incineration. Thus, a preliminary assessment demonstrated that the net GHGs emissions directly from waste treatment could be mitigated to 0.06–0.10 tCO_2_e/t MSW, significantly lower than the national average of 0.47–0.69 tCO_2_e/t MSW in 2018.

Elaborate research is to be conducted to investigate future waste flows under the circular economy paradigm and to evaluate its carbon neutrality potential. Reutilization of recyclable materials, reclamation of nutrients from organic waste and recovery of energy from incinerators can create both resource saving and carbon mitigation benefits and should be coherently counted across the country. Furthermore, a circular economy solution is needed to restore and regenerate aging waste landfills and dumps. 197 ± 44 Mt of carbon deposit in historic landfills must be safely treated or recovered, and the risks be minimized.

### Supplementary Information

Supplementary Information 1.

Supplementary Information 2.

## Data Availability

All data generated or analysed during this study are included in this published article and its supplementary information files.

## Abbreviations

CC: Central China
DOC: Degradable organic carbon
FoC: Fossil carbon
FOD: First-order decay model
GHGs: Greenhouse gases emissions
IPCC: Intergovernmental Panel on Climate Change
MSW: Municipal solid waste
NC: North China
NE: Northeast China
NGE: Net GHGs emissions
NW: Northwest China
nFoC: Non-fossil carbon
SC: South China
SE: Southeast China
SW: Southwest China
TC: Total carbon content
