# Supplementary material for: Tracking the carbon flows in municipal waste management in China
Source: Sci Rep. 2024 Jan 17;14:1471. doi: 10.1038/s41598-024-51698-0 (PMC10794192; doi:10.1038/s41598-024-51698-0)
Supplement: Supplementary file 1 — Supplementary Information 1. [file 41598_2024_51698_MOESM1_ESM.pdf]

Supplementary File for:

Tracking the carbon flows in municipal waste management in China

Jing Zhang [1,2], Huanzheng Du [1,4], Tao Wang [2,3,4], Peiyuan Xiao [3,4], Sha Lu [1,3], Gang Zhao [3,5], Jianfu Zhao [3], Guangming Li [3]

[1] Circular Economy Research Institute, School of Marxism, Tongji University, 1239 Siping Rd., Shanghai, 200092, China.

[2] Institute of Carbon Neutrality, Tongji University, 1239 Siping Rd., Shanghai, 200092, China.

[3] College of Environmental Science and Engineering, Tongji University, 1239 Siping Rd., Shanghai, 200092, China.

[4] UNEP-Tongji Institute of Environment for Sustainable Development, Tongji University, 1239 Siping Rd., Shanghai, 200092, China.

[5] Shanghai Urban Construction Design & Research Institute Groups Co., Ltd., 3447 Dongfang Rd., Shanghai, 200120, China.

A list supplementary information is provided as below.

(1) Table S1. Variables to compute carbon flows in MSW.

(2) Table S2. Composition of municipal solid waste (MSW) in cities of China.

(3) Table S3. Average water content in MSW in China.

(4) Table S4. Default content of DOC, fossil carbon, and total carbon in MSW, according to IPCC (2019).

(5) Table S5. Coefficients of SGompertz regression of total carbon content vs. GDP per capita.

(6) Table S6. Coefficients of SGompertz regression of DOC vs. GDP per capita.

(7) Table S7. Per capita carbon flows and GHGs emissions from MSW in Chinese provinces.

(8) Fig. S1. Regression of carbon content in MSW vs. GDP per capita.

(9) Fig. S2. Relationship between total carbon content and retail sales per capita.

(10) Fig. S3. Relationship between total carbon content and consumption expenditure per capita.

(11) Fig. S4. Relationship between total carbon content and food expenditure per capita

(12) Fig. S5. Relationship between DOC and retail sales per capita.

(13) Fig. S6. Relationship between DOC and consumption expenditure per capita.

(14) Fig. S7. Relationship between DOC and food expenditure per capita

(15) Fig. S8. Cumulative carbon flows in MSW in China in 2000, values are in MtC. FoC, fossil carbon; nFoC, non-fossil carbon.

(16) Fig. S9. Cumulative carbon flows in MSW in China in 2010, values are in MtC. FoC, fossil carbon; nFoC, non-fossil carbon.

(17) Fig. S10. Cumulative carbon flows in MSW in China in 2018, values are in MtC. FoC, fossil carbon; nFoC, non-fossil carbon.

(18) Fig. S11. Net GHGs emissions ratio of China in 2000-2018.

The values, definitions, and notes of variables in equation (1) to equation (14) are illustrated in Table S1.

Table S1. Variables to compute carbon flows in MSW.

Variable	Definition	Value	Note
c_t	The total carbon content in gross MSW		Compared with the DOC, the carbon in rubber and plastic is attached. It is calculated based on Eq. (15).
c_o	The aggregate DOC percentage in gross MSW		It is calculated based on Eq. (15).
c_f	The fossil carbon content in gross MSW		It is calculated based on Eq. (15).
dc_L	Decomposed proportion of DOC during landfills	50%	IPCC, 2006.
dc_D	Decomposed proportion of DOC during simple landfills and dumping	50%	IPCC, 2006.

dc_{CO2}	Decomposed proportion of CO_2 during composting	60%	When MSW completely rotten, more than 99.5% biodegradable organic content is decomposed (Garcia et al., 1992). In the composting process, about 60% of carbon will be converted to CO_2. (Sommer and Moller, 2000, Beck-Friis et al., 2001, Wolter et al., 2004, El Kader et al., 2007, Szanto et al., 2007).
dc_{CH4}	The proportion of CH_4 in in composting	5%	In the composting process, about 5% of carbon will be converted to CH_4. (Sommer and Moller, 2000, Beck-Friis et al., 2001, Wolter et al., 2004, El Kader et al., 2007, Szanto et al., 2007).
r_{CH4}	Landfills gas recovery rate	23%	Landfills gas recovery ratio. (Zhan and Wang, 2022).
f_{CH4}	Landfill gas flaring rate	7%	Landfills gas flaring ratio. (Zhan and Wang, 2022).
mcf_L	Methane correction factors of sanitary landfills	100%	The default value of methane correction factor based on the method of disposal in MSW sanitary landfills.
mcf_D	Methane correction factors of simple landfills and dumping	60%	The default value of methane correction factor based on the method of disposal in MSW simple landfills and dumping.
μ_L	The proportion of CH_4 in landfills gas	50%	IPCC, 2006.
μ_D	The proportion of CH_4 in simple landfills and dumping gas	50%	IPCC, 2006.
ox	Methane oxidation factor in landfills	10%	IPCC, 2006.
δ	Oxidation factor in incineration	95%	IPCC, 2006.

MSW composition was obtained from literature and waste sampling. Major data and their sources are displayed in Table S2.

Table S2. Composition of municipal solid waste (MSW) in cities of China.

Year	Province	City	Plastic %	Textile %	Wood %	Paper %	Food waste %	Source
2007	Anhui	Hefei	13.6	3.96	0.66	3.26		Wu et al., 2008.
2018	Beijing	Beijing	18.9	0.85	4.11		51.4	Cheng, 2020.
2012	Fujian	Xiamen	13.0	5.60			67.0	Xiao et al., 2016.
2006	Gansu	Lanzhou	11.3	2.10	1.36	9.69	37.2	Ji, 2007.
2012	Guangdong	Dongguan	17.0	0.40	0.24	16.5	59.5	Fang and Zeng, 2013.
2011	Guangdong	Foshan	28.0	3.00	3.50	6.50	45.0	Zhang et al., 2015.
2015	Guangdong	Guangzhou	21.0	3.99	4.51	13.0	51.0	Lv, 2020.
2011	Guangdong	Shenzhen	12.9	10.2		12.4	50.7	Luo, 2006.
2010	Guizhou	Anshun	5.46	5.88	3.89	6.86		Chen et al., 2014.
2001	Hainan	Haikou	9.60	1.19		6.33	50.6	Luo et al., 2004.
2005	Hebei	Shijiazhuang	5.97	1.61	0.97	3.07	62.7	Su et al., 2008.
2006	Hebei	Tangshan	10.8	2.20		2.20	46.2	Wang et al., 2007.
2010	Henan	Zhengzhou	10.8	2.20	41.5	3.90		Song and Wang, 2012.
2008	Heilongjiang	Harbin	7.40	1.31	72.1	9.02		Sun, 2016.
2000	Hubei	Wuhan	7.27	1.25	1.68	5.62	47.7	Liu and Jiang, 2001.
1998	Jilin	Jilin	10.6	1.15	2.79	7.82	35.6	Liu et al., 1999.
2007	Jiangsu	Nanjing	12.0	1.00		9.00	66.0	Kang and Wang, 2009.
2007	Jiangsu	Suzhou	18.6	4.18	0.86	10.9	62.6	He et al., 2008.
2017	Jiangxi	Jiujiang	18.8	15.4	11.3	13.7	21.6	Fan et al., 2018
2003	Liaoning	Dalian	18.6	1.98	36.2	8.76	1.27	Zhao, 2006a.
2011	Liaoning	Shenyang	10.6	3.10	9.20			Zhou et al., 2013.
2001	Inner Mongolia	Huhhot	9.20	0.30	0.40	6.50	32.0	Zhao et al., 2005.
2012	Shandong	Jinan	9.92	3.04	0.95	11.2	58.7	Li and Zheng, 2014.
2001	Shanxi	Datong	3.09	0.77		2.98	44.2	Lian, 2004.

2018	Shaanxi	Xi'an	19.6	2.30	2.50	13.5	57.5	Liu et al., 2019.
2018	Shanghai	Shanghai	22.9	2.8	3.2	12.7	53.6	Actual measurement
2014	Sichuan	Chengdu	15.4		14.8		34.8	Fu et al., 2014.
2013	Sichuan	Dazhou	9.05	0.70	1.01	14.5	60.0	Liu, 2015.
2013	Sichuan	Barkam	10.8	3.52	2.38	10.1	51.4	Liu, 2015.
2010	Sichuan	Mianyang	21.9	3.60	1.01	10.3	60.2	Huang and Liu, 2012.
2013	Sichuan	Nanchong	10.4	1.78	4.00	16.5	55.3	Liu, 2015.
2010	Sichuan	Neijiang	16.0	0.70	0.89	9.00	64.0	Huang and Liu, 2012.
2010	Sichuan	Panzhihua	15.6	1.00	3.10	8.20	57.4	Huang and Liu, 2012.
2010	Sichuan	Suining	11.0	4.30	1.40	8.00	68.1	Huang and Liu, 2012.
2013	Sichuan	Xichang	7.94	0.11	4.01	12.3	45.2	Liu, 2015.
2013	Sichuan	Yibin	7.22	2.22	4.34	11.6	50.0	Liu, 2015.
2012	Tianjin	Tianjin	14.6			11.7	63.2	Peng et al., 2014.
2011	Tibet	Lhasa	14.8	4.50	18.5	23.7		Dian and Bu, 2012.
2009	Xinjiang	Shihezi	9.60	2.50		5.60	59.0	Chen et al., 2010.
2017	Xinjiang	Urumqi	21.3	8.37	11.7	21.6	14.6	Zhang, 2019.
2001	Yunnan	Kunming	5.13	1.85	7.15	2.68	37.5	Zhang et al., 2004.
2000	Yunnan	Lijiang	8.00			15.0	45.0	Wang, 2003.
2004	Zhejiang	Hangzhou	5.71	4.00	46.2	6.66		Zhou, 2010.
2011	Zhejiang	Jiaxing	5.52	1.45	0.77	8.3	53	Actual measurement
2016	Zhejiang	Ningbo	15.7	22.8	2.04	20.9	50.6	Chen et al., 2018.
2006	Chongqing	Chongqing	11.8	2.84	1.53	5.39	24.4	Li et al., 2007.

The moisture content is shown in Table S3.

Table S3. Average water content in MSW in China.

MSW component	Water content (% gross weight)	Sources
Food waste	66.1	Chen et al., 2018.<br>Zhao, 2006.
Paper	44.3	Yang et al., 2018.<br>Chen and Liu, 2010.
Textile	40.8	Fang and Zeng, 2013.<br>Fan et al., 2018.
Plastic	37.7	Lian, 2004.<br>Liu et al., 1999.
Wood	35.6	Su et al., 2008.<br>Ji, 2007.

For DOC and fossil carbon content in waste dry weight, the default values of the IPCC (2019) guidelines are applied (See Table S4).

Table S4. Default content of DOC, fossil carbon, and total carbon in MSW, according to IPCC (2019).

Composition	DOC (% dry waste)	Fossil carbon (% dry waste)	Total carbon (% dry waste)
Food waste	38	0	38
Wood	50	0	50
Paper	44	2	46
Textile	20	30	50
Plastic	0	75	75

The regression results are presented in Table S5 and Table S6.

Table S5. Coefficients of SGompertz regression of total carbon content vs. GDP per capita.

a		x_c		*k*		Statistics	
Value	Standard Error	Value	Standard Error	Value	Standard Error	Reduced Chi-Sqr	Adj. R-Square
26.796	6.0104	-1.4218	0.9835	0.1280	0.0739	4.0119	0.6392

Table S6. Coefficients of SGompertz regression of DOC vs. GDP per capita.

a		x_c		*k*		Statistics	
Value	Standard Error	Value	Standard Error	Value	Standard Error	Reduced Chi-Sqr	Adj. R-Square
12.797	1.2600	-3.2905	1.8668	0.1961	0.1092	1.1816	0.4986

Table S7. Per capita carbon flows and GHGs emissions from MSW in Chinese provinces.

Province	Carbon per cap in 2000 (kgC/cap)	Carbon per cap in 2018 (kgC/cap)	Net Emission per cap in 2000 (kgCO2/cap)	Net Emission per cap in 2018 (kgCO2/cap)	Deposited Carbon per cap in 2000 (kgC/cap)	Deposited Carbon per cap in 2018 (kgC/cap)
Beijing	36.67	128.28	150.31	288.58	25.05	57.07
Tianjin	46.16	56.18	189.63	150.29	31.57	23.61
Hebei	46.44	34.28	197.16	99.49	31.66	13.92
Shanxi	68.08	41.25	237.17	147.33	46.27	22.06
Inner Mongolia	41.40	51.75	168.72	172.71	28.10	28.64
Liaoning	61.00	58.35	240.07	219.42	41.51	38.11
Jilin	79.46	75.47	316.99	219.90	54.11	39.09
Heilongjiang	65.15	44.25	249.56	154.18	44.34	25.52
Shanghai	67.25	115.25	281.04	249.77	46.44	52.73
Jiangsu	26.30	70.15	121.28	144.89	17.93	14.21
Zhejiang	15.05	68.75	66.64	159.19	10.09	19.04
Anhui	34.49	48.70	139.33	110.91	23.35	12.30
Fujian	23.37	74.52	103.12	167.76	16.04	20.16

Jiangxi	23.06	31.86	97.21	117.49	15.65	17.12
Shandong	45.91	72.31	211.50	161.98	31.30	20.08
Henan	33.85	33.72	151.94	128.77	22.97	19.24
Hubei	39.57	56.27	173.34	161.54	26.91	24.09
Hunan	25.37	70.32	102.44	222.19	17.25	32.89
Guangdong	30.51	89.32	136.28	262.63	20.95	40.60
Guangxi	17.46	33.43	78.44	116.73	11.84	16.21
Hainan	50.59	116.26	228.50	286.27	34.44	35.86
Chongqing	22.29	50.67	100.24	153.34	15.10	20.17
Sichuan	31.02	58.16	121.27	157.63	20.64	19.92
Guizhou	36.94	33.65	131.97	105.83	25.05	14.91
Yunnan	24.68	35.85	107.54	95.32	16.90	11.67
Tibet	77.11	98.57	250.82	272.95	52.03	35.60
Shaanxi	26.44	54.40	102.37	239.88	17.98	38.32
Gansu	41.53	39.05	169.59	121.75	28.17	17.27
Qinghai	67.58	67.81	268.00	242.85	45.72	47.15
Ningxia	58.73	54.32	223.41	165.78	39.95	23.08
Xinjiang	64.62	70.15	202.34	254.76	44.38	48.95

Multiple regressions have been tested such as carbon content with consumption expenditure per capita and total retail sales of consumer goods (Fig. S1-S7). GDP per capita was probably the most feasible indicator for a nonlinear regression.

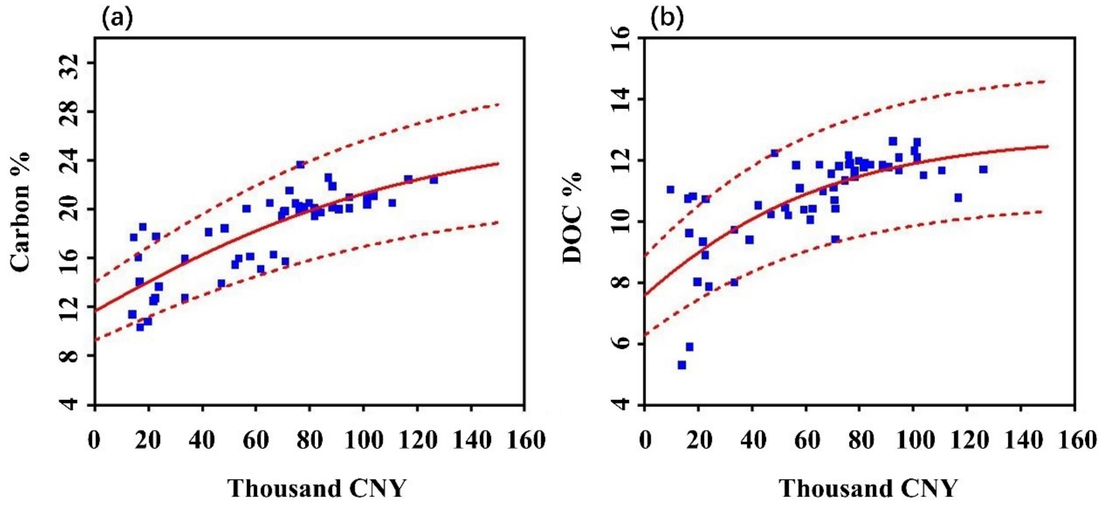

Fig. S1. Regression of carbon content in MSW vs. GDP per capita.

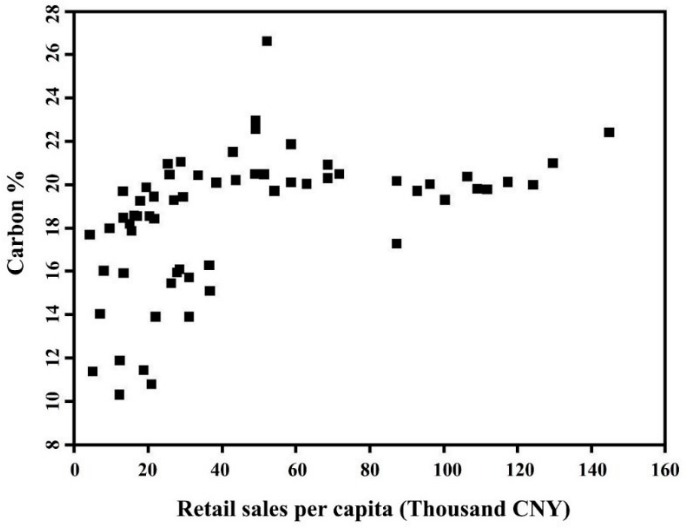

Fig. S2. Relationship between total carbon content and retail sales per capita.

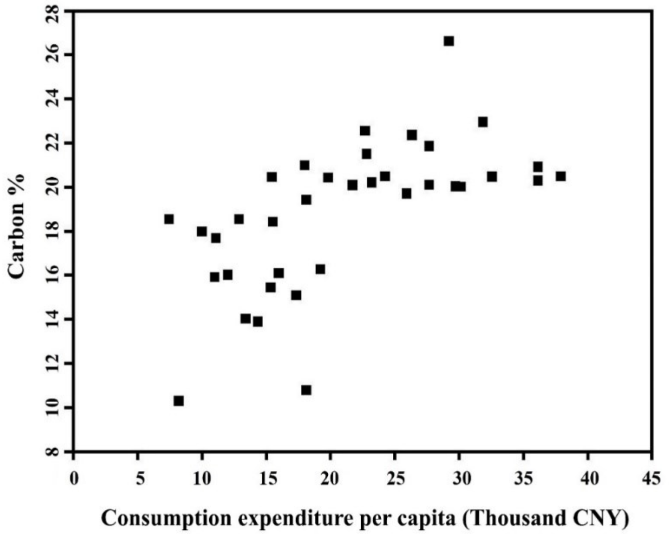

Fig. S3. Relationship between total carbon content and consumption expenditure per capita.

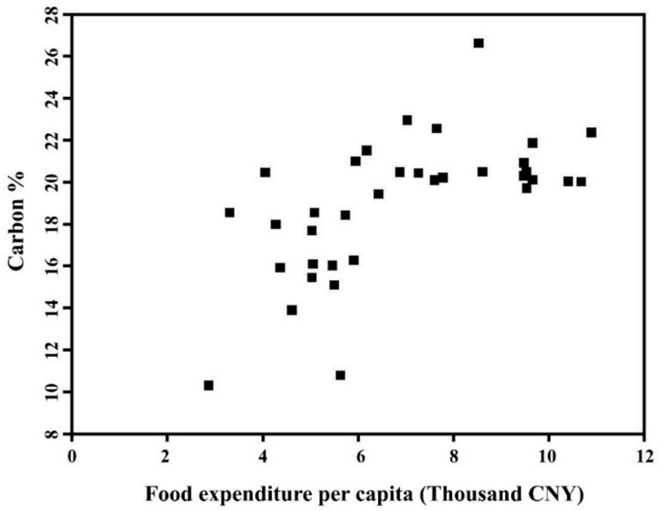

Fig. S4. Relationship between total carbon content and food expenditure per capita.

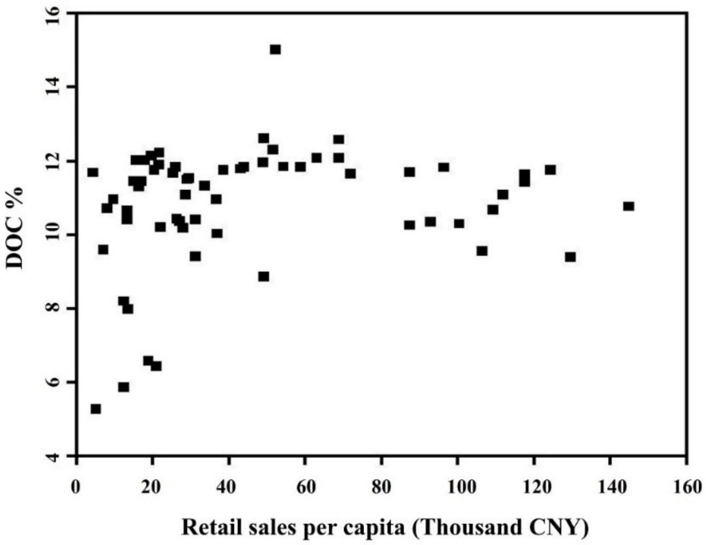

Fig. S5. Relationship between DOC and retail sales per capita.

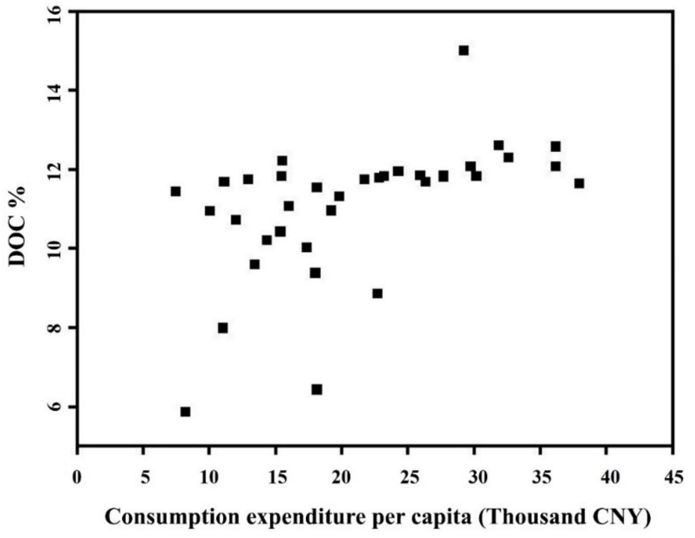

Fig. S6. Relationship between DOC and consumption expenditure per capita.

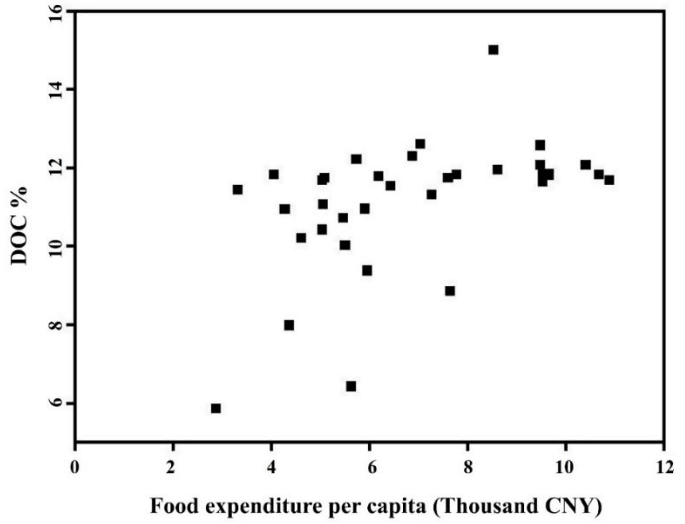

Fig. S7. Relationship between DOC and food expenditure per capita.

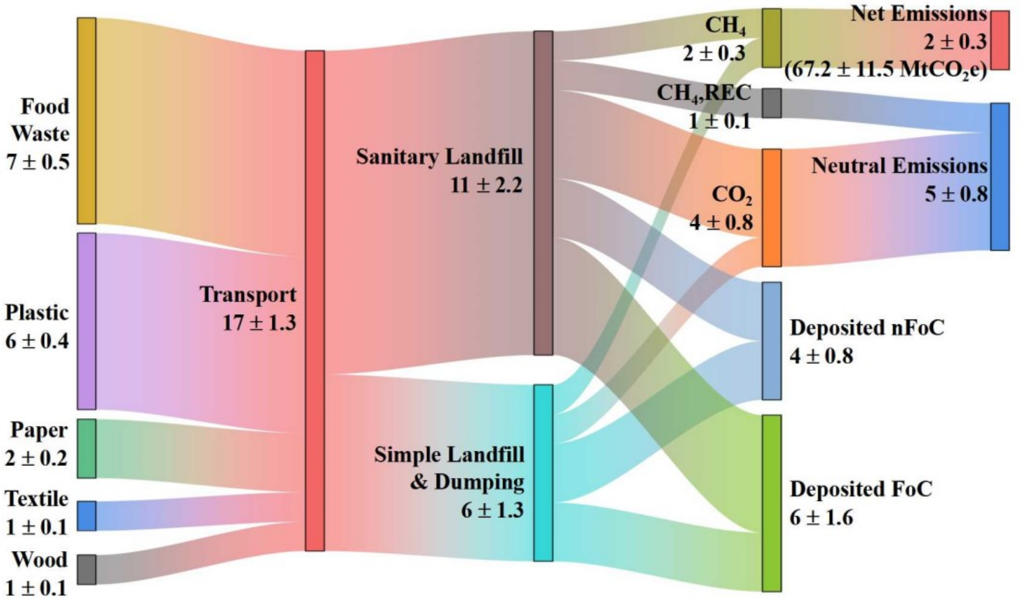

Fig. S8. Cumulative carbon flows in MSW in China in 2000, values are in MtC.

FoC, fossil carbon; nFoC, non-fossil carbon.

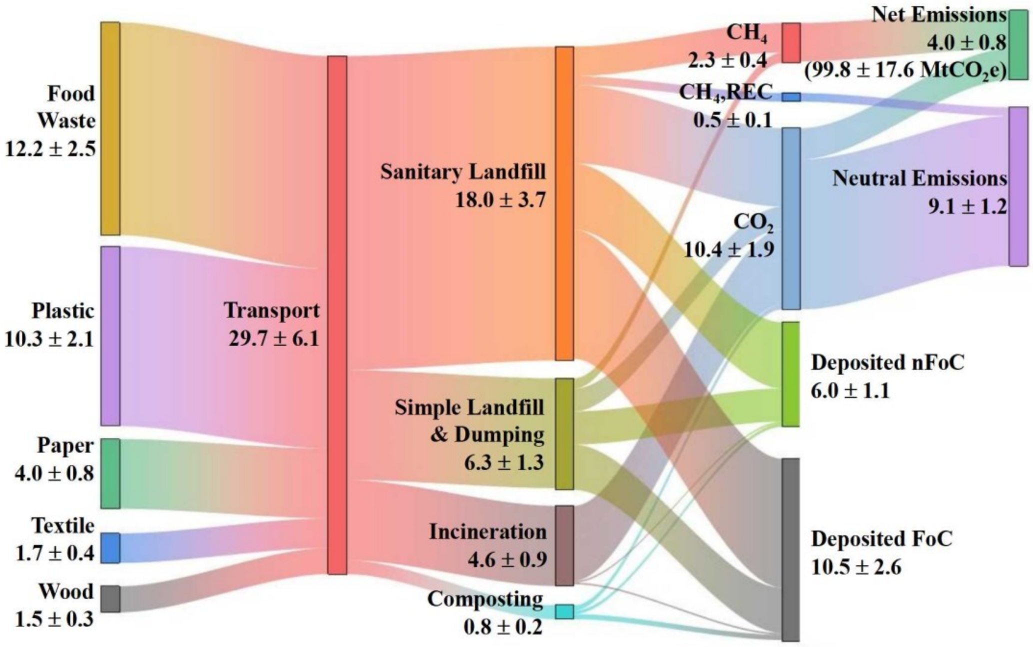

Fig. S9. Cumulative carbon flows in MSW in China in 2010, values are in MtC.

FoC, fossil carbon; nFoC, non-fossil carbon.

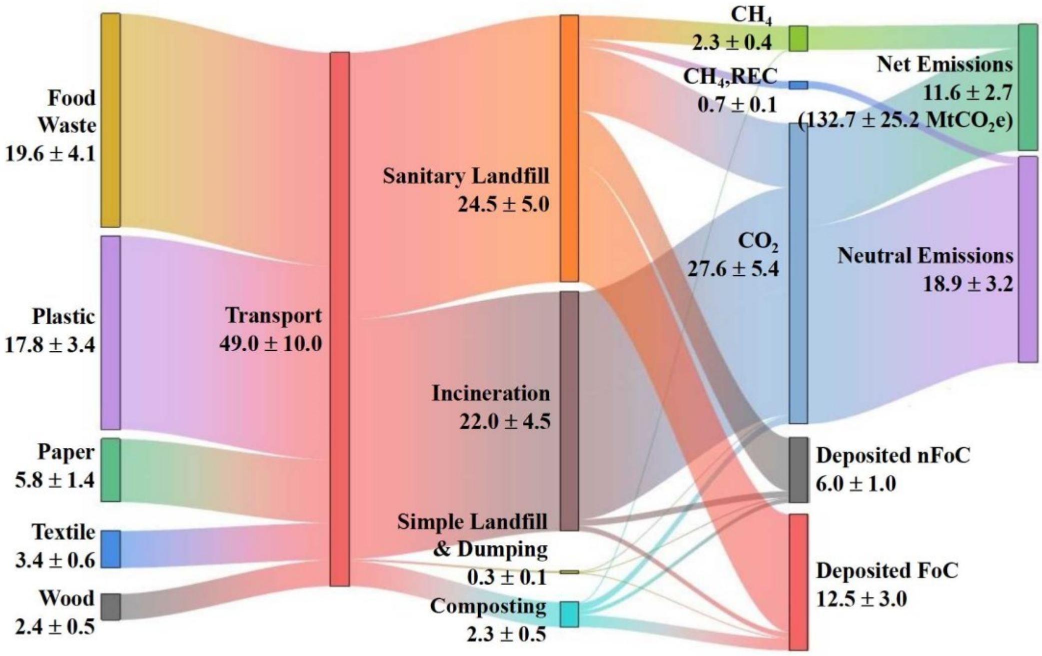

Fig. S10. Cumulative carbon flows in MSW in China in 2018, values are in MtC. FoC, fossil carbon; nFoC, non-fossil carbon.

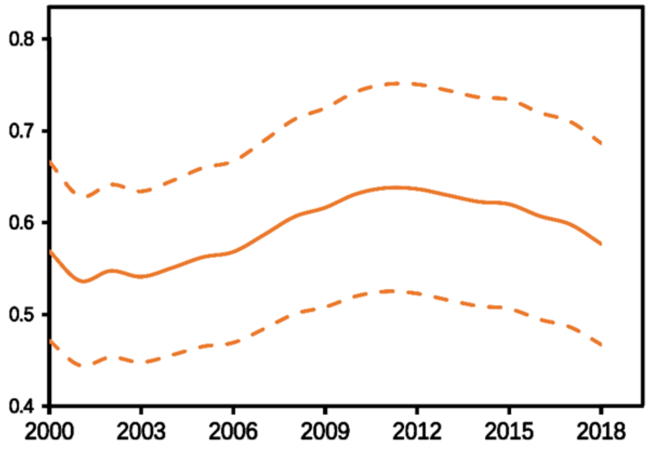

Fig. S11. Ratio of net GHGs emissions to MSW generation (tCO_2e/t) in China in 2000-2018

References

Beck-Friis B., Smars S., Jonsson H, et al. Gaseous emissions of carbon dioxide, ammonia and nitrous oxide from organic household waste in a compost reactor under different temperature regimes. *J Journal of Agricultural Engineering Research*,,**78(4)**: 423-430. (2001)

Chen, C., Wei, C. & Wang, Z. Garbage composition and four water parameters of the leachate from the landfill in Anshun city. *J. Journal of Guizhou University (Natural Science Edition)* **31(3)**, 124-128. (in Chinese) (2014).

Chen, H., Lei, K., Ma, C. & Gao, S. Analysis on constituent and physical and chemical characteristics of MSW in Shihezi. *J. Journal of Anhui Agricultural Sciences* **38(23)**, 12666-12668. (in Chinese) (2010).

Chen, J. & Liu, D. Heat value monitoring of Xiamen domestic waste and analysis of its influencing factors. *J. Environmental Sanitation Engineering* **18(6)**: 14. (in Chinese) (2010).

Chen, Q., Yang, D., Huan, Y., Deng, M. & Yang, X. Composition and physicochemical properties of classified municipal solid waste in different districts of Ningbo city. *J. Acta Scientiae Circumstantiae* **38(3)**, 1064-1070. (in Chinese) (2018).

Cheng, W. Comparative analysis of the composition characteristics of domestic waste in Beijing urban and rural areas. *J. Renewable Resources and Circular Economy* **13(1)**, 17-22. (in Chinese) (2020).

Dian, Z. & Bu, D. Analysis on physical characteristics of domestic waster in the urban area in Lhasa. *J. Journal of Tibet University (Natural Science Edition)* **27(1)**,

20-27. (in Chinese) (2012).

El Kader N. A., Robin P., Paillat I. M., et al, Turning, compacting and the addition of water as factors affecting gaseous emissions in farm manure composting. *J. Bioresource Technology*, **98(14)**:2619-2628. (2007)

Fan, R., Gan, Z., Xiao, G. & Wang, T. Analysis of composition and caloric value of municipal solid waste of one city in East China. *J. Guangdong Chemical Industry* **45(373)**, 40. (in Chinese) (2018).

Fang, C. & Zeng, C. Study on domestic garbage characteristics and pilot testing of household registration system. *J. Environmental Science and Management* **38(6)**, 12-15. (in Chinese) (2013).

Fu, Y., Qiu, Z., Fu, C., Zhu, D. & Yin, Y. Analysis of municipal solid waste composition and physicochemical properties in urban area of Chengdu. *J. Sichuan Environment* **33(6)**, 126-129. (in Chinese) (2014).

Garcia, C., Costa, H.F. & Ayuso, M. Evaluation of the maturity of municipal waste compost using simple chemical parameters commun. *J. Soil Science and Plant Analysis* **23 (13/14)**, 1501-1512 (1992).

He, S., Zhu, S. & Yu, L. Characteristic analysis and treatment countermeasures of domestic waste in Suzhou city. *J. Environmental Sanitation Engineering* **16(6)**, 62-64. (in Chinese) (2008).

Huang, M. & Liu, D. Characteristics and composition of municipal solid waste in Sichuan province. *J. Environmental Monitoring in China* **28(5)**, 121-123. (in Chinese) (2012).

Ji, A. The analysis of physical characteristics and incinerating feasibility of municipal solid waste in Lanzhou (Doctoral dissertation, Lanzhou: Lanzhou University). (in Chinese) (2007).

Kang, X. & Wang, S. Survey and minimization analysis of municipal domestic waste of Nanjing city. *J. Environmental Science and Management* **34(2)**: 46-48. (in Chinese) (2009).

Li, L., Zheng, X. & Mao, K. Status quo and countermeasures of domestic waste treatment in the main urban area of Chongqing. *J. Energy Conservation* **26(5)**, 49-51. (in Chinese) (2007).

Li, M. & Zheng, Z. Property change of domestic waste and diversified transition of treatment technologies in Jinan. *J. Environmental Sanitation Engineering* **22(4)**, 62-64. (in Chinese) (2014).

Lian, J. The status quo and countermeasures of the garbage problem in Datong city. *J. Shanxi Energy and Conservation* **(1)**, 24-25. (in Chinese) (2004).

Liu, B., Wu, M. & Zhang, L. Municipal solid waste characteristics of Jilin city (Doctoral dissertation). *J. Joural of Northeast China Institute of Electric Power Engineering* **19(3)**: 16. (in Chinese) (1999).

Liu, D. & Jiang, D. Analysis of staple element changes of urban domestic waste in Wuhan. *J. Environmental Sanitation Engineering* **9(4)**, 173-176. (in Chinese) (2001).

Liu, Y. Characteristics and heavy metal analysis of municipal waste Sichuan. *J. Sichuan Agricultural University* (in Chinese) (2015).

Liu, Y. et al. Analysis of composition of municipal solid waste in Xi'an and anaerobic fermentation performance with different solid concentrations. *J. China Biogas* **37(6)**, 37-43. (in Chinese) (2019).

Luo, T. Experimental research on the MSW properties and selection of seperation plan in Bao'an district of Shenzhen (Doctoral dissertation, Wuhan: Huazhong University of science and Technology). (in Chinese) (2006).

Luo, T., Ouyang, Z., Wang, X. & Li, W. Carbon output through urban domestic garbage Haikou (Doctoral dissertation). *J. Environmental Science* **25(6)**: 154. (in Chinese) (2004).

Lv, Y. Analysis of the model for estimating calorific value of municipal solid waste in South China. *J. China Resources Comprehensive Utilization* v.**38**; No.398(01), 88-91. (in Chinese) (2020).

Peng, W. et al. Physical composition and influencing factors of municipal domestic waste in Tianjin. *J. Environmental Sanitation Engineering* **3**. (in Chinese) (2014).

Sommer S. G., Moller H. B. Emission of greenhouse gases during composting of deep litter from pig production-effect of straw content. *J. Journal of Agricultural Science*, **134(5)**: 327-335. (2000)

Song, H. & Wang, T. Status and countermeasures of domestic waste treatment in Zhengzhou city. *J. Environmental Sanitation Engineering* **20(3)**, 28-32. (in Chinese) (2012).

Su, Y., Xing, S. & Wang, H. Management, disposal status and countermeasures

of domestic waste in Shijiazhuang city. *J. Environment and Health: The Environment and Health Forum of Hebei Society of Environmental Sciences and the Proceedings of the 2008 Academic Annual Conference*. (in Chinese) (2008).

Sun, G. Harbin municipal waste treatment and disposal status and management. *J. Heilongjiang Science and Technology Information* **(10)**, 298-298. (in Chinese) (2016).

Szanto G. L., Hamelers H. M., Rulkens W. H., et al. NH, NO and CH, emissions during passively aerated composting of straw-rich pig manure. *J. Bioresource Technology*, **98(14)**: 2659-2670. (2007)

Wang, H. Research on treatment of city domestic refuse in Lijiang county. *J. Yunnan Environmental Science* **22(3)**, 59-61. (in Chinese) (2003).

Wang, Y., Dong, X., Zhang, Y. & Zhang, S. An analysis of features of urban consumer waste in Tangshan city. *J. Journal of Tangshan College* **20(6)**, 62-63. (in Chinese) (2007).

Wolter M., Prayitno S., Schuchardt F., Greenhouse gas emission during storage of pig manure on a pilotscale. *J. Bioresource Technology*, **95(3)**: 235-244. (2004)

Wu, K., Yu, Z., Jin, J., Cai, J. & Chu, W. Discussion on the selection of Hefei municipal solid waste treatment plan. *J. Journal of Hefei University (Natural Science Edition)* **18(3)**, 56-58. (in Chinese) (2008).

Xiao, L., Ye, Z. & Lin, T. Analysis of household waste generation and its driving pattern based on community classification. *J. Journal of Environmental Sciences* **36(1)**, 307-313. (in Chinese) (2016).

Yang, N., Shao, L. & He, P. Study on the moisture content and its features for municipal solid waste fractions in China. *J. China Environmental Science* **38(3)**: 1033-1038. (in Chinese) (2018)

Zhan, JF., Wang, SS. Assessment of Methane Generation and Relevant Carbon Emission from MSW Landfills in China. *J. Environmental Science & Technology* **45 (11)**:147-155. (in Chinese) (2022).

Zhang, H., Li, X., Qi, J., Chen, Y. & Fang, J. Analysis of composition characteristics of municipal solid waste in South China. *J. Environmental Science* **(1)**, 325-332. (in Chinese) (2015).

Zhang, J., Xie, G. & Su, C. Study on treatment of municipal domestic refuse in Kunming. *J. Yunnan Environmental Science* **23(1)**, 3-5. (in Chinese) (2004).

Zhang, P. Physical composition and characteristics of domestic waste in Urumqi. *J. Regional governance.* (in Chinese) (2019).

Zhao, J., Sun, W., Yang, J. & Jiang, Q. Analysis of the composition and characteristics of municipal solid waste in Hohhot. *J. Acta Scientiarum Naturalium Universitatis NeiMongol (Natural Science Edition)* **36(1)**, 100-103. (in Chinese) (2005).

Zhao, W. Survey and analysis of municipal domestic waste in central area of Dalian city. *J. Environmental Sanitation Engineering* **14(6)**, 29-31. (in Chinese) (2006).

Zhou, F. Research on comprehensive evaluation method of Hangzhou municipal solid waste treatment system (Doctoral dissertation, Changsha: Central South

University). (in Chinese) (2010).

Zhou, X., Xu, J., Li, Y. & Li, R. Study on scheme of domestic waste energy utilization model in Shenyang city. *J. Efficient and Clean Energy Utilization and New Energy Technology-Proceedings of the 2012 Power Engineering Youth Academic Forum*. (in Chinese) (2013).
